# NDVI dynamics under changing meteorological factors in a shallow lake in future metropolitan, semiarid area in North China

**DOI:** 10.1038/s41598-018-33968-w

**Published:** 2018-10-29

**Authors:** Yunlong Zhang, Xuan Wang, Chunhui Li, Yanpeng Cai, Zhifeng Yang, Yujun Yi

**Affiliations:** 1 State Key Laboratory of Water Environment Simulation, Beijing Normal University, Beijing, 100875 China; 2 Key Laboratory for Water and Sediment Sciences of Ministry of Education, Beijing Normal University, Beijing, 100875 China; 3 Beijing Engineering Research Center for Watershed Environmental Restoration and Integrated Ecological Regulation, School of Environment, Beijing Normal University, Beijing, 100875 China

## Abstract

Three meteorological parameters, including one parameter representing water conditions (i.e., precipitation) and two parameters representing energy conditions (i.e., net radiation and air temperature), were used to make an in-depth analysis of the response of Normalized Difference Vegetation Index (NDVI) dynamics to climate change in Lake Baiyangdian, a shallow lake located in Xiong’an New Area (XNA), a future metropolitan in North China. The results showed that the vegetation coverage of the entire area remained at a medium level with average NDVI being 0.46 during 2000–2015. At a yearly scale, water was the key factor controlling the reed growth in Lake Baiyangdian. NDVI variations in each season had different water/energy driving factors. In spring, summer and autumn, vegetation growth was mainly affected by net radiation, air temperature and air temperature, respectively. Time-lags between NDVI and the meteorological parameters varied from parameters and seasons. Taken together, this research broadened our cognition about response characteristics of NDVI dynamics to water and energy variations through adding an important meteorological parameter (i.e., net radiation). With the rapid construction of XNA, it could be helpful for accurately understanding impacts of climate change on vegetation growth and be beneficial for effective ecosystem management in water shortage areas.

## Introduction

Vegetation, as one of the key components of any terrestrial ecosystem, plays a fundamental role in regulating water cycle, energy exchange and carbon cycles^1,2^. It is recognized as overt evidence of biological responses to multiple environmental factors^3,4^. Climate change as a crucial environmental factor has resulted in significant effects on vegetation dynamics, and it has been considered as one of the most important driving factors in structure and function variations of ecosystem^5–10^. Using modern telemetry technology (e.g., remote sensing technology) to investigate vegetation growth dynamics and its influencing factors are the foundation to ensure ecosystem health at regional or the global scales^11^. As an indicator closely related to green biomass and leaf area indices at regional or global scales, the Normalized Difference Vegetation Index (NDVI) has been widely recognized for studying the changes in terrestrial vegetation pattern and the ability for vegetation to absorb photosynthetically active radiation^12–15^. In recent years, study on responses of NDVI dynamics to water and energy variations in different seasons has been paid more attention for effective ecosystem management^16^.

Many researchers attempted to reveal the correlation between NDVI and meteorological parameters. Normally, air temperature and precipitation were the most widely used parameters, and many studies demonstrated the mechanism effects of air temperature and precipitation on plant growth^4,16–24^. Actually, there are some other meteorological parameters also affecting vegetation succession and development should not be ignored. For example, evapotranspiration is an important parameter of the water resource management, water requirement and vegetation growth^25,26^. And the Penman-Monteith (PM) formulation recommended by the Food and Agriculture Organization of the United Nations is regarded as a good evapotranspiration estimator for a wide variety of climatic conditions^27^. In the PM formula, net radiation is an indispensable climatic parameter determining evapotranspiration^28^. It also represents important energy input for terrestrial ecosystems^22,29–31^. Net radiation is the balance between incoming and outgoing energies at the earth’s surface or the balance between the absorbed, reflected and emitted energies by the earth. It is an important parameter for evapotranspiration and provide energy for supporting vegetation growth^19,27,31^. However, in previous studies, relationships analysis between NDVI and net radiation had seldom been discussed. Thus, it hinders comprehensive understanding of the relationship between vegetation statuses and changing climatic conditions, and inevitably affects ecosystem adaptive management decisions. Through considering an important energy parameter (i.e., net radiation) besides conventional parameters, the research will broaden and deepen the analysis about response of NDVI dynamics to changing climatic conditions.

As for the relationship between NDVI and meteorological parameters, another important issue is time lags resulting from the delayed response of vegetation growth to climate change. Analyzing time lags between NDVI and meteorological factors is critical to develop a strategy of the crop planting, management and ecosystem protection under climate change^32^. In general, previous studies results were not always consistent. Many research suggested that there was a 20–40 day lag between NDVI and meteorological parameters^33^. Comparatively, a number of research showed that there was an approximate 2–3 month lag^34^. At the same time, they calculated time lags taking different seasons as a whole for conventional research and lacked comparisons of time lag in different seasons^16,33–35^. Whether time lags in different seasons are consistent should be further discussed. Thus, in order to comprehensively understand the impact of climate condition changes on vegetation growth statuses, the time lag analyses between NDVI and meteorological parameters in different seasons are necessary.

A millennium plan of establishing Xiong’an New Area, abbreviated XNA (i.e., a future metropolitan in North China), for the development of science and technology culture in Beijing-Tianjin-Hebei or even in whole China, was announced by the Chinese government on April 1^st^, 2017^36–38^. The central government hopes XNA to become a livable and environmentally friendly city. The largest freshwater lake of the northern China plain, Lake Baiyangdian, is referred to as the Kidney of North China and located in the territory of XNA. It is also a water scarce wetland in the semi-arid area of northern China. Partly because of a large amount of natural evapotranspiration and artificial water withdrawal, its ecological environment is fragile. In particular, the recent construction of dams and reservoirs upstream of the lake, which are intended to provide economic and social benefits to water users, has profoundly changed the natural hydrologic regime^22,39^. Low water use efficiency and ecological degradation will hinder the implementation of the plan of establishing XNA. Wang *et al*.^22,30^ investigated correlation between NDVI dynamics and many meteorological variables in Lake Baiyangdian, including precipitation, sunshine, air temperature and so on, broadening hydro-climatological influencing factors of vegetation growth compared to previous literature. However, they did not consider net radiation and neglected to analyze the lagging response of NDVI to variations of meteorological factors. Therefore, under the effects of both water scarce and human activities, it is crucial to grasp the relationship between NDVI dynamics and water/energy conditions for making decisions associated with the promotion of water use efficiency and ecological health in such a water shortage area^5,40^.

Therefore, the objective of the present study is to give an in-depth investigation on how and why vegetation change has occurred under water and energy variations in Lake Baiyangdian. Firstly, the trends of NDVI and meteorological parameters about water and energy will be examined in the study area over the past 16 years (from 2000 to 2015). Secondly, through wavelet transform coherence and the Pearson correlation, correlations between NDVI and meteorological parameters including two aspects of water and energy will be investigated in different seasons. Finally, using the lag correlation coefficient, time lags between NDVI and meteorological parameters will be analyzed in different seasons. This research can link ecosystem development to climate change more precisely through extending important meteorological parameter (i.e., net radiation) that was neglected in previous studies, and update our understanding about correlations between NDVI dynamics and changing climatic conditions. It would be helpful to provide an effective decision-making support for adaptive ecosystem management under climate change.

## Results and Discussion

### Temporal variations of NDVI and meteorological parameters

**Figure 1:**
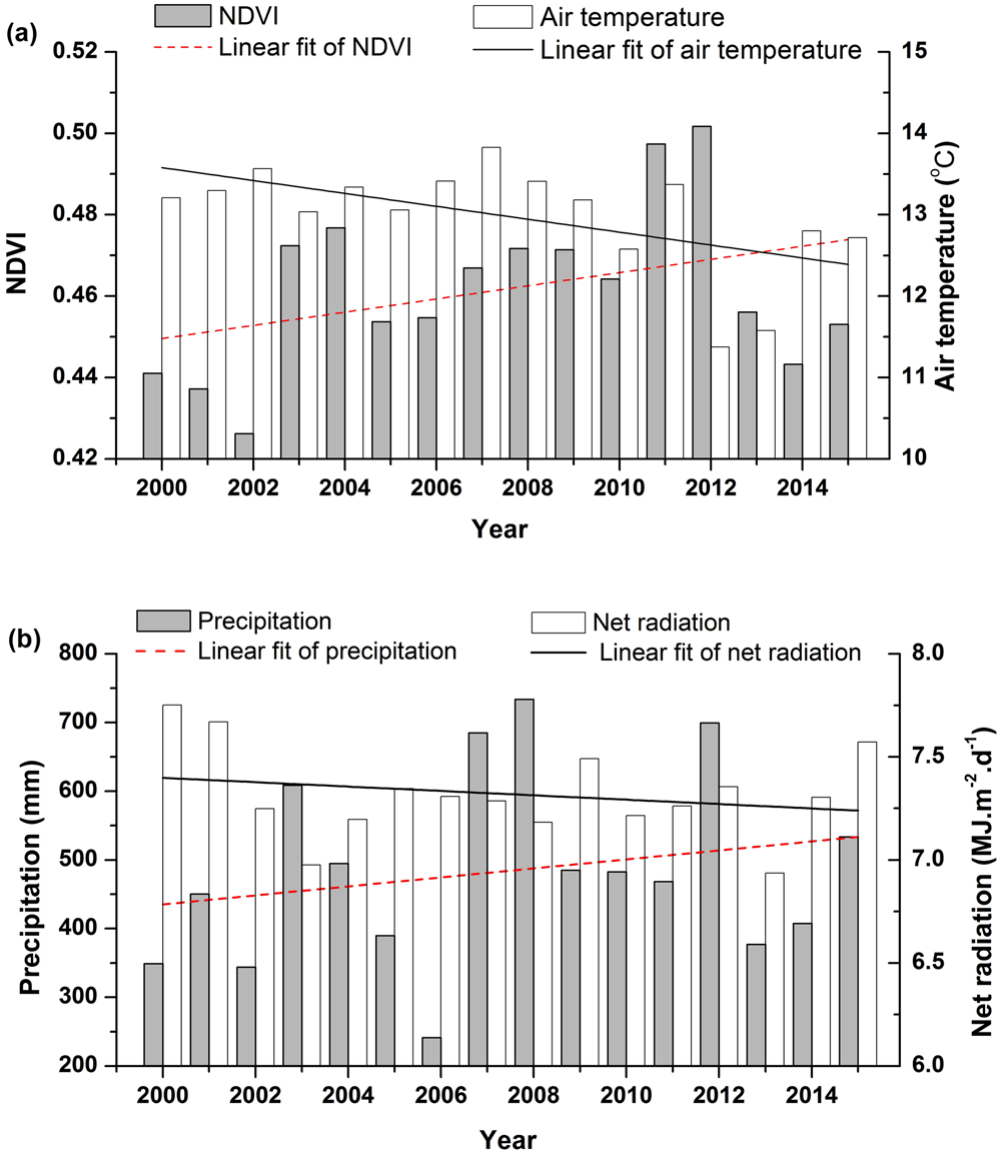
Inter-annual variations of NDVI and meteorological parameters of water and energy in Lake Baiyangdian from 2000 to 2015. (**a**) Annual averaged NDVI and annual averaged air temperature; (**b**) Annual cumulative precipitation and annual averaged net radiation.

During the period of 2000–2015, the annual averaged NDVI of Lake Baiyangdian showed a slight increasing trend (not significant), it reached the maximum (0.502) in 2012 and the minimum (0.426) in 2002 (Fig. 1). In general, NDVI had been fluctuating, and the main reason may be that many times ecological water replenishment since 2000 and water replenishment is helpful to plant growth^41^. The vegetation coverage of the whole area remained at a medium level, and the average NDVI was 0.46 from 2000 to 2015. Meteorological parameters also showed continuously fluctuating in this period. The air temperature and net radiation of Lake Baiyangdian showed a declined trend (not significant), while the precipitation showed a slight increasing trend (not significant). The annual average air temperature, net radiation and annual cumulative precipitation were 12.98 °C, 7.32 KJ·m^−2^·d^−1^ and 484.37 mm. Although the variation trends of NDVI and meteorological parameters were not significant (NDVI increase, water factors increase and energy parameters decrease), previous studies showed that precipitation was the main factor determining vegetation growth in arid and semi-arid regions. In the years with heavy rainfall, the vegetation grows lush; and in the years with low rainfall, the growth slows down and the vegetation becomes sparse^6,42^. Li *et al*. showed that excessive air temperature could limit vegetation growth^43^. This is because the increase of air temperature will promote the vegetation transpiration, leading to the lack of available water for vegetation growth, especially in arid and semi-arid regions. In conclusion, the characteristics of variations in NDVI and meteorological parameters exhibited dynamic and nonlinear.

**Figure 2:**
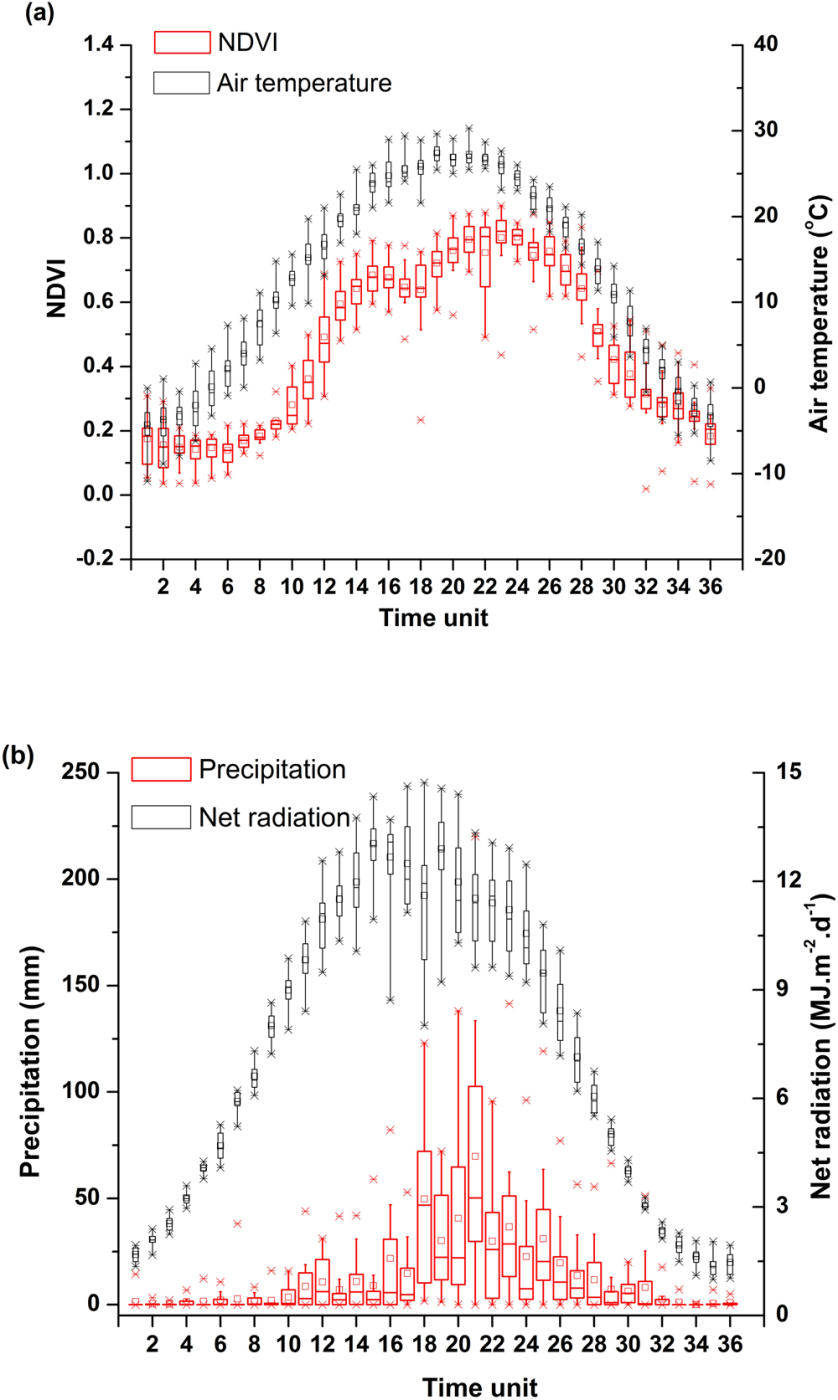
Boxplots of 10-day variations in NDVI and meteorological parameters of water and energy in Lake Baiyangdian. (**a**) 10-day NDVI and 10-day air temperature; (**b**) 10-day precipitation and 10-day net radiation.

In order to more clearly examine the NDVI trend during the year, Fig. 2 shows the variations of NDVI and meteorological parameters in 10-day intervals over the period of 2000–2015 (i.e., January 1 to 10 is the 1^st^ time unit; December 20 to 31 is the 36^th^ time unit). Limited by the energy and water conditions, the 10-day NDVI had a remarkable increase from the 10^th^ time unit (April) to the 24^th^ time unit (August). However, during the period of April to August, there was a marked decrease from 15^th^ time unit to 17^th^ time unit. This is agreement with the results from Guo *et al*.^35^, who found that NDVI had a marked decrease from June to August. The maximum NDVI value was 0.803 in the 24^th^ time unit (August). After showing the annual maximum value in late August, NDVI had a continuous decrease until November to the same levels of those in spring. From November to the next April, NDVI is very low, the minimum NDVI value was 0.14 in the 6^th^ time unit (February).

Although the 10-day NDVI had a similar pattern to that of the climate variables, the 10-day peak NDVI was not always coupled with those climatic factors (Fig. 2). Among the 36 time units, the largest air temperature, precipitation and net radiation were seen in the 19^th^ unit, the 21^st^ unit and the 15^th^ unit. These time units instead of the 24^th^ unit with the largest NDVI, this discrepancy may suggest a lagged response of NDVI to change in meteorological parameters. This could partly be due to the fact that the dynamic process of plant growth, the vegetation needs time to respond to the changes in the environment.

### Relationship between NDVI and meteorological parameters at a yearly scale

**Figure 3:**
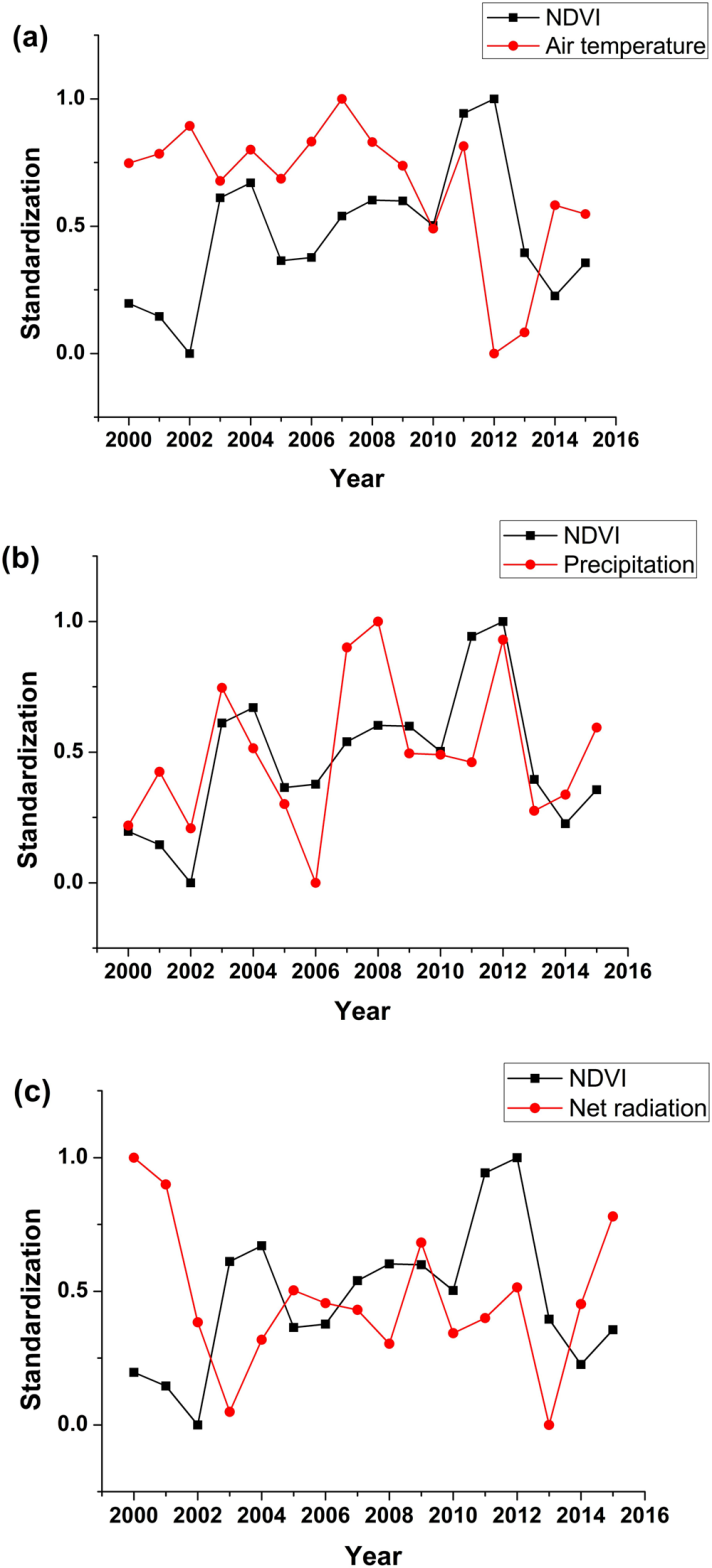
The relationship between the normalized NDVI and normalized meteorological parameters of water and energy in Lake Baiyangdian at a yearly scale from 2000 to 2015. (**a**) NDVI and air temperature; (**b**) NDVI and precipitation; and (**c**) NDVI and net radiation.

**Figure 4:**
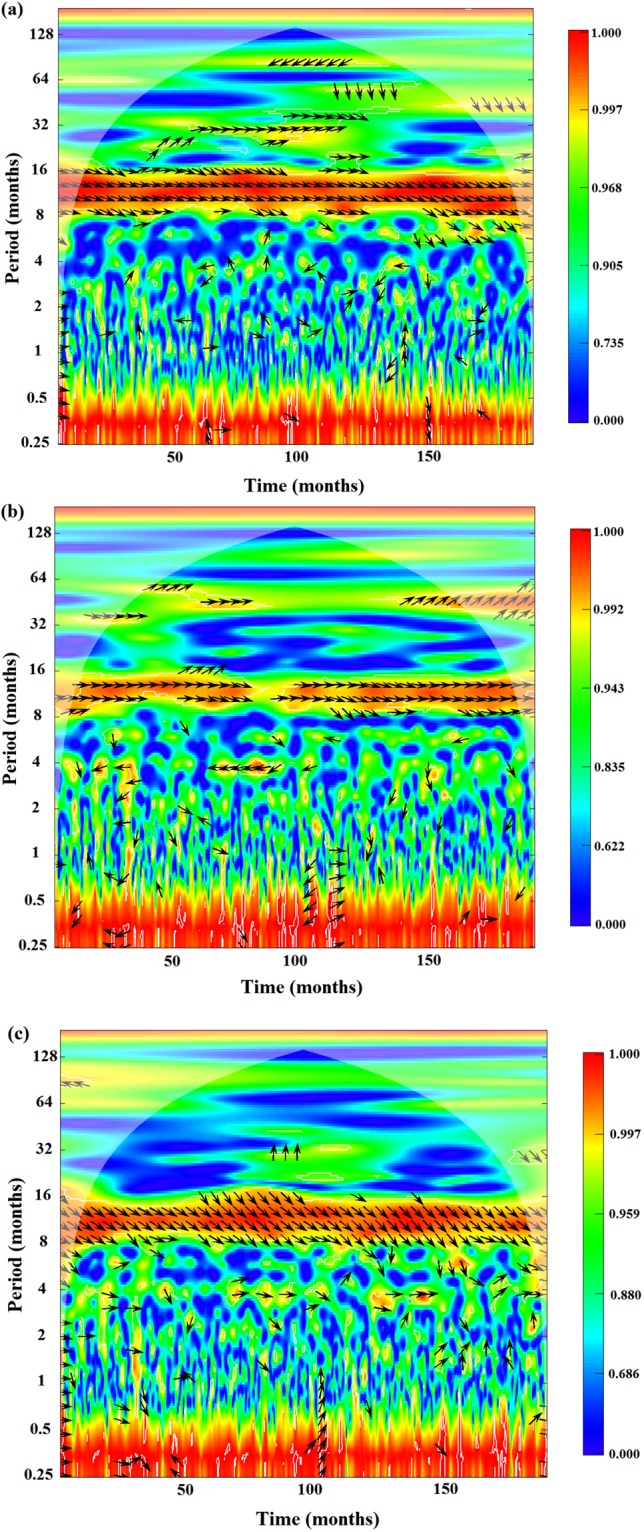
Wavelet coherence transforms of NDVI with meteorological parameters. (**a**) Air temperature; (**b**) Precipitation; and (**c**) Net radiation.

With normalization method, the normalized relationships between NDVI and meteorological parameters of water and energy were analyzed at a yearly scale (Fig. 3). Climate change (especially precipitation change) has influences on NDVI, and the change patterns of NDVI are always similar with those of precipitation. NDVI decreased with reduced precipitation and increased with increased precipitation (Fig. 3(c)). To further analyze the relationships between NDVI and meteorological parameters, we used the wavelet coherence transforms which can provide a reliable basis for period, correlation and lag analysis (Fig. 4). The ribbon indicates the magnitude of the wavelet coherence at a specific time and frequency (i.e., the period). The arrows indicate the phase difference between the meteorological parameters and NDVI patterns (i.e., the lag). The arrows are plotted only within white contour lines indicating significance (with respect to the null hypothesis of white noise processes) at the 10% confident level. The results showed that the major resonance periods between meteorological parameters and NDVI equaled approximately 8–16 months during 2000–2015. NDVI was closely correlated with air temperature, precipitation and net radiation. Annual fluctuations in meteorological parameters coincided with those in the NDVI values. In addition, high peaks of coherence were observed at lower time-scales (scales between 0.25 and 0.5 month), even though there were not significant at the 10% confident level. According to the phase angle (showed by the arrows), there were time lags between NDVI and the meteorological parameters. Time lags of NDVI to air temperature, precipitation and net radiation were 1 month, 0 month and 1.5 months, respectively (Table 1). In general, the time lag of NDVI to energy parameters (i.e., air temperature and net radiation) was longer than water parameters (i.e., precipitation) in Lake Baiyangdian. This is generally consistent with other literatures, which demonstrated that there were time lags between NDVI and climatic factors^12,31,35,44^.

**Table 1:** The characteristics of wavelet coherence transforms of NDVI with meteorological parameters.

Parameters	Major resonance period/month	Significant time/a	Phase angle/rad	Time lags/month
Air temperature	8–16	2000–2015	0.52	1.0
Precipitation	8–16	2000–2015	0	0
Net radiation	8–16	2000–2015	0.79	1.5

For some a certain meteorological factor, the corresponding lag time or the response speed of vegetation variations can reflect the requirement of vegetation growth for it. The above results showed that the time lag of NDVI to energy parameters (i.e., air temperature and net radiation) was longer than water parameter (i.e., precipitation). The main reason may be attributed to the study area is located in a semi-arid area with small annual rainfall and high annual evapotranspiration. Between 2000 and 2015, the water level of Lake Baiyangdian was lower than the universally accepted ecological water level (i.e., 6 m) many times, and even became the dry lake. The low water content was not conducive to replenishing groundwater reserves and fail to provide continuous water supply to the vegetation. Therefore, water factors had a bigger restriction on vegetation growth than energy factors, indicating NDVI had a higher sensitivity to water parameters. For this reason, the time lag of NDVI to water parameter (i.e., precipitation) was shorter than energy parameters (i.e., air temperature and net radiation).

The quantitative correlations between NDVI and meteorological parameters of water and energy were analyzed subsequently using the Pearson correlation coefficient and partial correlation coefficient, as shown in Eq. (10). The results showed that only precipitation had significant positive correlations with NDVI (*p* < 0.01), and other three meteorological parameters exhibited negative correlations with NDVI (not significant) (Table 2). The main reason of negative correlation may be attributed to these parameters were positively correlated with evapotranspiration^45–47^, leading to an acceleration in the transpiration of plants and evaporation of the surface, which inhibited the growth of vegetation. These findings suggested that the main driver of NDVI variations was precipitation, water may be the key factor controlling the reed growth in Lake Baiyangdian at a yearly scale.

**Table 2:** Pearson correlation coefficients and partial correlation coefficients between NDVI and meteorological parameters of water and energy at a yearly scale in Lake Baiyangdian.

Period	Correlation coefficients			Partial correlation coefficients		
	R_NDVI-T_	R_NDVI-P_	R_NDVI-N_	PR_NDVI-T_	PR_NDVI-P_	PR_NDVI-N_
2000–2015	−0.282	0.601*	−0.313	−0.234	0.585*	−0.220

Note: *represents P < 0.05 and **represents P < 0.01 significance; R_NDVI-T_, R_NDVI-P_ and R_NDVI-N_ represent correlation coefficients between NDVI and air temperature (°C), NDVI and precipitation (mm), and NDVI and net radiation (MJ·m^−2^·d^−1^), respectively; and PR_NDVI- T_, PR_NDVI- P_ , and PR_NDVI-N_ represent partial correlation coefficients between NDVI and air temperature (°C), NDVI and precipitation (mm), and NDVI and net radiation (MJ·m^−2^·d^−1^), respectively.

### Relationship between NDVI and meteorological parameters in different seasons

**Figure 5:**
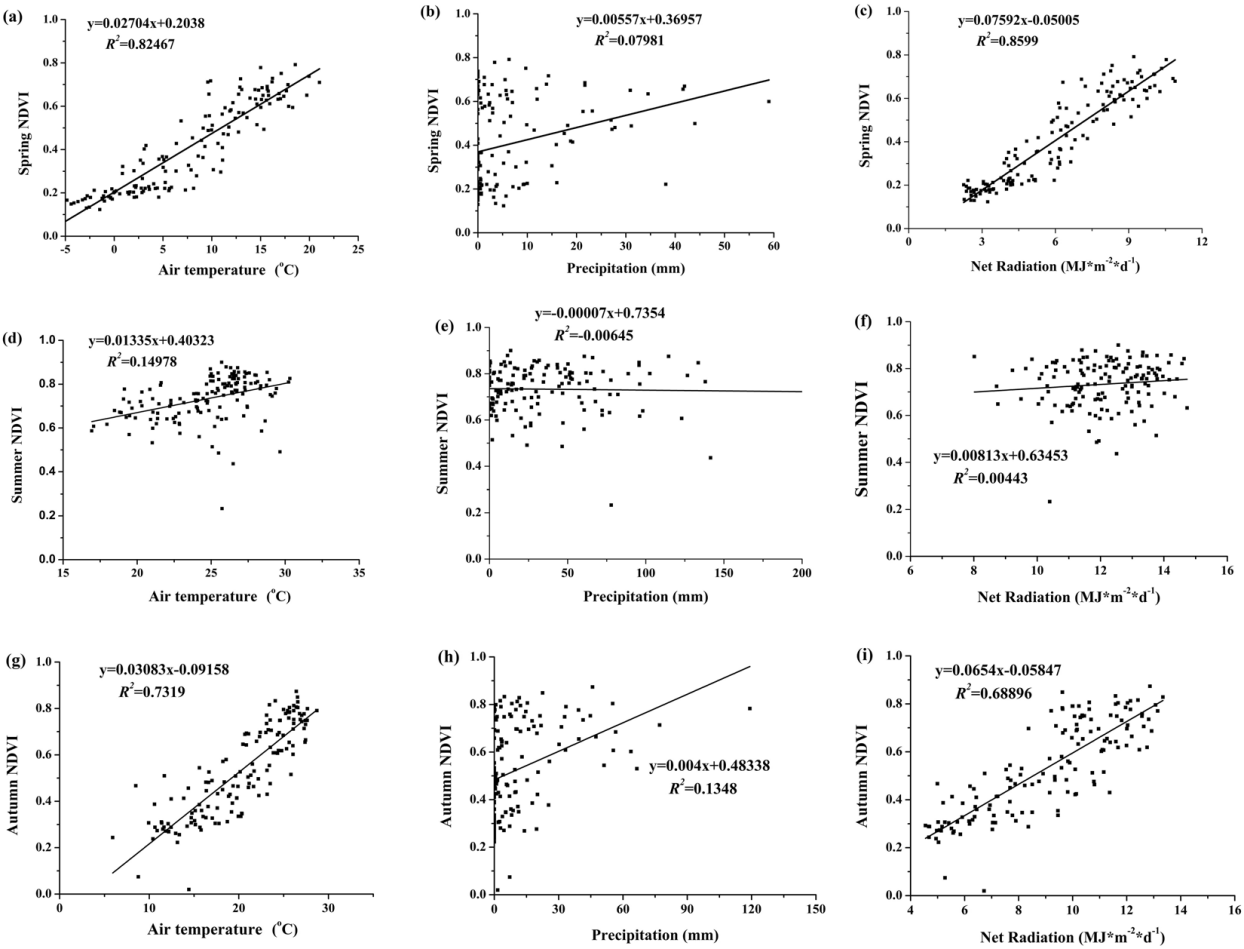
Seasonal response of NDVI to water and energy parameters at a 10-day scale in Lake Baiyangdian. (**a**–**c**) represent relationships between NDVI and air temperature, precipitation and net radiation respectively, in spring; (**d**–**f**) represent relationships between NDVI and air temperature, precipitation and net radiation respectively, in summer; and (**g**–**i**) represent relationships between NDVI and air temperature, precipitation and net radiation respectively, in autumn.

With taking into account the differing time lags for each climate factor, the relationship between the NDVI and climate variables was analyzed in three seasons of plant growth separately (Fig. 5). Spring, summer and autumn were March to May, June to August and September to November, respectively. Table 3 shows that the partial correlation between NDVI and meteorological parameters from high to low were: net radiation > air temperature > precipitation in spring; air temperature > precipitation > net radiation in summer; and air temperature > net radiation > precipitation in autumn. In general, the response of NDVI to meteorological parameters showed that it had different driving factors of water/energy in each season in Lake Baiyangdian.

**Table 3:** Pearson correlation coefficients and partial correlation coefficients between NDVI and meteorological parameters of water and energy at a 10-day scale in three growing seasons.

Season	Correlation coefficients			Partial correlation coefficients		
	R_NDVI-T_	R_NDVI-P_	R_NDVI-N_	PR_NDVI-T_	PR_NDVI-P_	PR_NDVI-N_
Spring	0.909**	0.294**	0.928**	0.338**	0.069	0.548**
Summer	0.395**	−0.024	0.107	0.389**	−0.088	−0.009
Autumn	0.857**	0.375**	0.831**	0.414**	0.103	0.273**

Note: *represents P < 0.05 and **represents P < 0.01 significance; R_NDVI-T_, R_NDVI-P_ and R_NDVI-N_ represent correlation coefficients between NDVI and air temperature (°C), NDVI and precipitation (mm), and NDVI and net radiation (MJ·m^−2^·d^−1^), respectively; and PR_NDVI-T_, PR_NDVI-P_ , and PR_NDVI-N_ represent partial correlation coefficients between NDVI and air temperature (°C), NDVI and precipitation (mm), and NDVI and net radiation (MJ·m^−2^·d^−1^), respectively.

Comparatively, only precipitation had significant positive correlations with NDVI at yearly scale (Table 2), and the results at the 10-day scale were quite different (Table 3). The difference in driving factors of water/energy at large time scales (e.g., the yearly scale) and small time scales (e.g., the seasonal scale or the 10-day scale) implied that the correlation between NDVI and meteorological parameters was scale-dependent. This inconsistence in different scales could partly be due to the following fact: the main crop of Lake Baiyangdian, i.e., reed, was one of the world’s most widely distributed species; it can distribute in medium environment, wet environment and aquatic environment. According to its biological characteristics, reed required a large amount of water when it grew up. Thus, water was one of the most important factors affecting the growth and distribution of reeds^48^. In humid years with large rainfall, the vegetation grew vigorously; on the contrary, the vegetation became withered. Therefore, at the yearly scale, the reed growth was greatly affected by precipitation (i.e., NDVI had a higher correlation with precipitation than other meteorological factors), and the effects of other environmental factors were covered up. However, at the small time scale (e.g., the seasonal scale or the 10-day scale), the main influencing factors in different growth stages of reeds were closely related to their physiological and ecological characteristics. Spring as a rapid growth period of vegetation, NDVI was significantly and positively related to air temperature and net radiation (Table 3). The main driver of spring NDVI variations in Lake Baiyangdian was energy condition (i.e., net radiation) (Table 3), the rapid growth caused by increased energy was an important reason for the expansion of vegetation coverage. Thus, energy may be the key factor controlling plant growth during the early stage. In summer, air temperature had positive correlations with NDVI. However, these correlation levels were low (*R*^2^ = 0.15, 0.006 and 0.004 for air temperature, precipitation and net radiation, respectively), the main reason may be that vegetation growth was in a slowing status in summer, so the correlation between NDVI and climate change was declining. In autumn, due to the decreased air temperature, energy condition limited vegetation growth. Thus, energy condition (i.e., air temperature) was the most important climate factor in this region. In general, at the 10-day scale, precipitation was concentrated in summer with the large evapotranspiration, so the water level of the lake was stable within each season. Thus, precipitation had a less impact on vegetation growth in each season; that is, NDVI had a poor correlation with precipitation, and other meteorological factors (i.e., air temperature and net radiation) had good correlation with vegetation growth. It showed that precipitation was not the main influencing factor, and the effects of other environmental factors (i.e., air temperature and net radiation) were highlighted at small time scale.

### Time lags between NDVI and meteorological parameters in different seasons

With taking different seasons as a whole, the time lag in vegetation response to climate change has been widely observed in other regions^12,31,35,44^, and the time lags vary due to local environments. In Lake Baiyangdian, to illustrate and analyze the lagged response of vegetation growth in different seasons, the maximum correlation coefficients and corresponding time lags were calculated. Three seasons including spring, summer and autumn, and multiple time lags (lagging 0 to 90 days) were combined to calculate time lags. The correlation coefficients between NDVI and each meteorological parameter of water and energy with different lag durations in these seasons were shown in Table 4. In spring, the time response of NDVI to air temperature was about 30 days. The maximum correlation coefficient between NDVI and precipitation had a lag time of 20 days. The lag time for net radiation were 50 days. In summer, the correspondingly time lags between NDVI and air temperature, precipitation and net radiation were 60 days, 30 days, and 90 days, respectively. In autumn, the correspondingly time lags were 0 days, 10 days, and 0 days, respectively.

**Table 4:** Correlation coefficients between NDVI and meteorological parameters of water and energy with different lag durations in growing seasons.

Season	Parameters	Time lags/day									
		0	10	20	30	40	50	60	70	80	90
Spring	Air temperature	0.893	0.905	0.900	0.909	0.900	0.870	0.847	0.776	0.656	0.322
	Precipitation	0.294	0.369	0.376	0.368	0.254	0.224	0.149	0.133	0.133	0.169
	Net radiation	0.898	0.894	0.920	0.924	0.928	0.929	0.924	0.928	0.916	0.890
Summer	Air temperature	0.254	0.241	0.338	0.395	0.452	0.519	0.553	0.524	0.518	0.512
	Precipitation	−0.020	0.307	0.154	0.336	0.334	0.104	0.128	0.201	0.232	0.106
	Net radiation	0.041	−0.280	−0.070	−0.160	0.107	0.239	0.379	0.459	0.495	0.516
Autumn	Air temperature	0.888	0.874	0.865	0.857	0.848	0.809	0.753	0.617	0.385	−0.090
	Precipitation	0.375	0.390	0.347	0.367	0.368	0.344	0.311	0.240	0.039	−0.100
	Net radiation	0.898	0.880	0.880	0.865	0.831	0.774	0.683	0.558	0.449	0.353

Comparing three seasons, we found time lags in spring and autumn was shorter than those in summer, and time lags in autumn were the shortest. It demonstrated that the response speed of autumn and spring NDVI to water and energy conditions changes was faster than that of summer NDVI, and autumn NDVI was the most sensitive. The results were generally consistent with previous literature, which demonstrated that vegetation response was more sensitive to climate change in spring and autumn^49^. Compared with the air temperature rapid increase in spring, the decrease of air temperature and precipitation in autumn were also rapid. Because autumn is the end of vegetation growth, the decrease of air temperature and precipitation were more likely to lead the rapid wilting of vegetation than other seasons, resulting in vegetation growth in autumn being the most sensitive to water energy conditions.

In northern China plain, water resources are relatively shortage. In Lake Baiyangdian, it was necessary to constantly carry out ecological water supply when the water level was less than a certain standard (e.g., usually about 6 meters). According to the results obtained in this research, in order to increase water use efficiency and ensure the health of the ecosystem, the timing of ecological water supplement may be more appropriate in the autumn than that in other seasons, with a full consideration of differences in sensitivity of ecosystem to water factors among different seasons as mentioned above. Therefore, this research would be helpful to provide an effective decision-making basis for adaptive water resources management and ecosystem management under climate change, especially in water shortage areas.

## Conclusions

This research analyzed the temporal variations of NDVI and their relation with meteorological parameters about water and energy in Lake Baiyangdian (i.e., the kernel of XNA), a semi-arid region in North China. The methods included wavelet analysis, correlation analysis and lag analysis. The conclusions were drawn as follows.

1. From 2000 to 2015, the vegetation coverage of the Lake Baiyangdian remained at a medium level with average NDVI being 0.46.
2. The correlation between NDVI and meteorological parameters of water and energy was scale-dependent for a yearly scale and a 10-day scale. In spring, summer and autumn, vegetation growth were mainly affected by net radiation, air temperature and air temperature, respectively.
3. Time-lags between NDVI and the meteorological parameters varied from parameters and seasons. Except autumn, the time lag of NDVI to energy parameters (i.e., air temperature and net radiation) was longer than water parameters (i.e., precipitation). The response speed of autumn and spring NDVI to water and energy conditions changes was faster than that of summer NDVI, and autumn NDVI were the most sensitive.

Compared with the conventional research that focused on some a single scale, it enriched the understanding about NDVI response to water and energy variations with an overall perspective with multiple time-scales. Compared to previous studies that analyzed time lags between NDVI and meteorology parameters with taking different seasons as a whole^33,34,44^, our research analyzed time lags in different seasons. The detailed time lags analyses can help us to precisely understand the response characteristics of vegetation to climate factors. Under adverse meteorological conditions, this research was useful for the adaptive management of a complex system of regional water resources and ecosystem, especially in water shortage areas. However, although this study would greatly improve understanding of the complicated responses of vegetation to climate changes, it could not distinguish the contribution rate of each meteorological factor. Other statistical methods, such as Random Forest (RF) and redundancy analysis (RDA) can be used in further study. RF can analyze the nonlinear relationship between meteorology and NDVI, and RDA can quantitatively analyze the contribution rate of different meteorological factors to NDVI changes for more precise findings. In the semiarid area, climate change, especially change in the water and energy meteorological factors, has played an important role in plant growth. Actually, besides decisive influences of changing meteorological factors (e.g., precipitation) on vegetation growth^50^, the nutrient contents of the lake or soil also have obvious influences. This would be considered in future research for effective environmental management.

## Materials and Methods

### Study area

**Figure 6:**
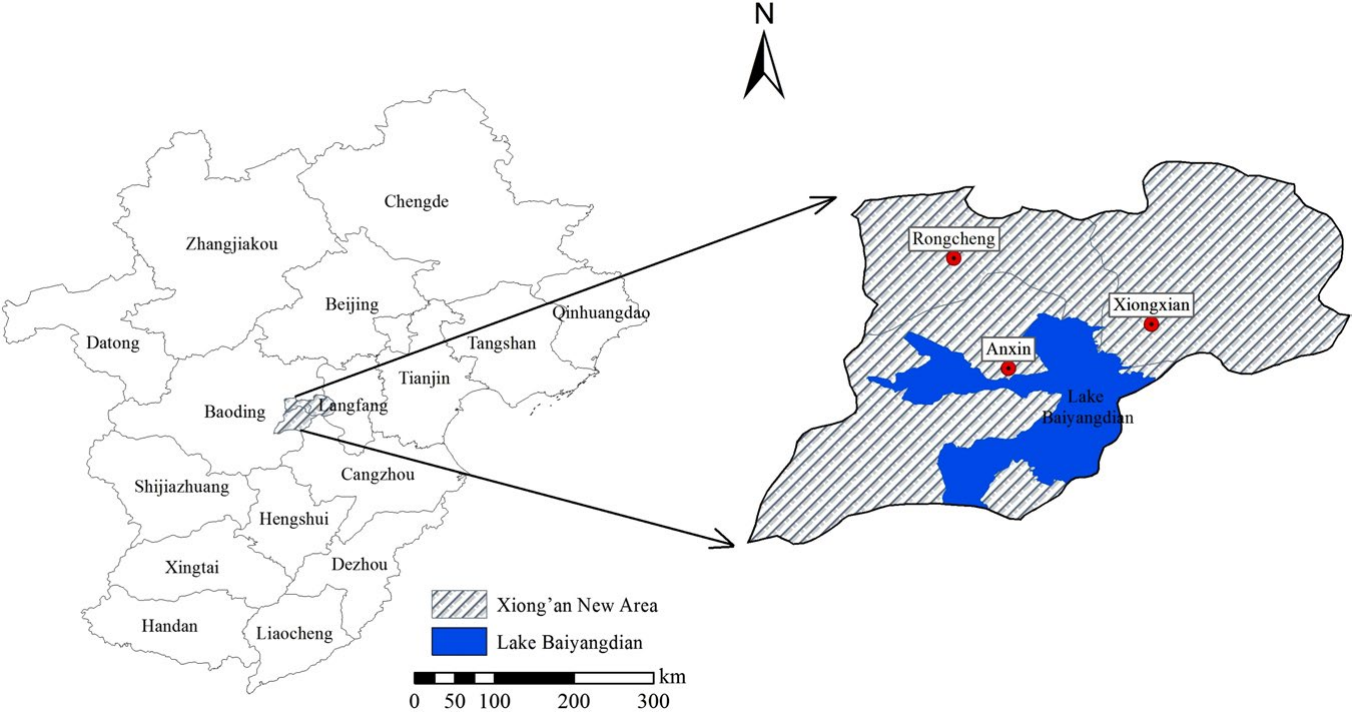
Geographical location of Lake Baiyangdian (the kernel of Xiong’an New Area) in North China.

Lake Baiyangdian (38°43′–39°02′N and 115°38′–116°07′E) in XNA (Fig. 6), is the largest freshwater lake in northern China plain. It lies in the middle reach of the Daqing River basin and discharges into the Bohai Gulf, Yellow Sea. The regional climate is warm temperate continental monsoons. Average annual air temperature is 7–12 °C, annual precipitation is 550 mm with 7–9 months contributing 80% of the total precipitation and the inter-annual amount of water fluctuates greatly. It is a grass-type shallow lake consisted of 143 islands and 67 km^2^ of reed marshes, its catchment is 31,200 km^2^. The lake surface area is 366 km^2^ and the average amount of water inflow is 908 million m^3^^22,30^. The predominant plants are natural low-lying depressions and reed marshes^51^. There are many villages around Lake Baiyangdian. Under the effects of both climate change and human activities, Lake Baiyangdian has faced a serious threat of ecological degradation.

### Data source

Vegetation change was identified using NDVI data derived from moderate-resolution imaging spectroradiometer dataset (MODIS), which has been widely used in studies about vegetation dynamics at regional and global scales^52–54^. The MODIS NDVI datasets of a 10-day interval for the period 2000–2015 were obtained from the International Scientific & Technical Data Mirror Site, Computer Network Information Center, Chinese Academy of Sciences (http://www.gscloud.cn/). The data calibrated to eliminate noise from atmospheric, cloud, aerosol, and sensor errors that were unrelated to vegetation growth^55^. To further eliminate the effects of clouds and filter aerosols, 10-day NDVI data was developed using the Maximum Value Composite (MVC) method^56^. The meteorological data were obtained from the National Meteorological Information Center (http://data.cma.cn/en). According to the distribution of weather stations in the National Meteorological Monitoring Network, only one weather station is close to Lake Baiyangdian. It is located 20 km north of Baiyangdian, and its station number is 54518 with the longitude and the latitude being 116.24E and 39.1N, respectively. The daily meteorological data, including average air temperature, maximum air temperature, minimum air temperature, vapor pressure, precipitation and sunshine duration, were obtained from the weather station. Then average air temperature, maximum air temperature, minimum air temperature and vapor pressure at 10-day scale were obtained by averaging the corresponding data within 10 days, and precipitation and sunshine duration at 10-day scale were obtained by cumulating corresponding data within 10 days. The characteristics of the data are as shown in Table 5.

**Table 5:** The characteristics of the data.

Parameter	Unit	Period	Time interval/spatial resolution
NDVI MODIS	—	March 2000 to December 2015	10 d /500 m
Average air temperature	°C	March 2000 to December 2015	10 d
Maximum air temperature	°C	March 2000 to December 2015	10 d
Minimum air temperature	°C	March 2000 to December 2015	10 d
Vapor pressure	kPa	March 2000 to December 2015	10 d
Precipitation	mm	March 2000 to December 2015	10 d
Sunshine duration	hour	March 2000 to December 2015	10 d

### Calculation of net radiation

Solar energy is the original energy source of the terrestrial ecosystem. When solar radiation penetrates the top of the atmosphere, energy comes into the atmosphere and goes out in two ways: reflection by clouds and aerosols. Finally, it arrives at the earth’s surface, but a considerable amount of the solar radiation reaching the earth’s surface will be reflected. Net radiation, also called net flux, is the balance between incoming and outgoing energy at the earth’s surface, or the balance between the energy absorbed, reflected and emitted by a horizontal plane. Since the albedo varies with surfaces, ecosystems usually gain different levels of net radiation even if they are bathed by the same amount of radiation^57–59^. In this research, net radiation (*R*_n_) was calculated using the method provided by the Food and Agriculture Organization of the United Nations:

1 $${R}_{n}={R}_{ns}-{R}_{nl}$$

where *R*_n_ is net radiation, MJ·m^−2^·day^−1^; *R*_ns_ is net solar radiation, MJ·m^−2^·day^−1^; and *R*_nl_ is net longwave radiation, MJ·m^−2^·day^−1^.

Net solar radiation (*R*_ns_) is the fraction of the solar radiation *R*_*S*_ (the amount of radiation reaching a horizontal plane) that is not reflected from the surface, and can be calculated using the following formula:

2 $${R}_{ns}=\frac{24\,(60)}{\pi }(1-\alpha )({a}_{s}+{b}_{s}\frac{n}{N}){G}_{sc}{d}_{r}[{\omega }_{s}\,\sin \,(\phi )\,\sin \,(\delta )+\,\cos \,(\delta )\,\sin \,({\omega }_{s})]$$

3 $${d}_{r}=1+0.033\,\cos \,(\frac{2\pi }{365}J)$$

4 $$\delta =0.409\,\sin (\frac{2\pi }{365}J-0.39)$$

5 $${\omega }_{s}=\arccos (-\tan (\phi )\,\tan (\delta ))$$

where *α* is the albedo or canopy reflection coefficient, which is 0.23 for a hypothetical grass reference crop^27^; *n* is the actual duration of sunshine, hours; *N* is the maximum possible duration of sunshine or daylight hours, hours; when n = 0, *a*_*s*_ is regression constant, expressing the fraction of extraterrestrial radiation reaching the earth on overcast days; when n = N, *a*_*s*_ + *b*_*s*_ is fraction of extraterrestrial radiation reaching the earth on clear days, and the values *a*_*s*_ = 0.25 and *b*_*s*_ = 0.50^27^; *G*_*SC*_ is the solar constant, here it is 0.0820 MJ·m^−2^·min^−1^ ^27^; *d*_*r*_ is the inverse relative distance from the earth to the Sun; *φ* is the latitude, rad; *δ* is the solar decimation, rad; *ω*_*s*_ is the sunset hour angle, rad; and *J* is the day in the year, between 1 to 365 or 366.

Net longwave radiation (*R*_*nl*_) represents the difference between outgoing and incoming longwave radiation that the earth’s surface emits or receives, and can be calculated as:

6 $${R}_{nl}=\sigma [\frac{{T}_{{\rm{\max }}\,{k}}^{4}+{T}_{{\rm{\min }}\,{k}}^{4}}{2}](0.34-0.14\sqrt{{e}_{a}})(0.1+0.9\,\frac{n}{N})$$

where *R*_*nl*_ is the net outgoing longwave radiation, MJ·m^−2^·day^−1^; *σ* is the Stefan-Boltzmann constant, here it is 4.903·10^−9^ MJ·K^−4^·m^−2^·day^−1^ ^27^; *T*_max *k*_ is the maximum absolute air temperature during the 24-hour period, degree Kelvin; and *T*_*min k*_ is the minimum absolute air temperature during the 24-hour period, degree Kelvin; and *e*_*a*_ is the vapor pressure, kPa.

### Parameters normalization

Since meteorological data and NDVI data are measured in different units of measurement, there was no comparability between these parameters. To achieve the comparison of these different parameters, the normalization was needed. Parameters can be normalized using Formula (7) to change them into unit-less parameters:

7 $${X^{\prime} }_{i}=\frac{({X}_{i}-{X}_{{\rm{\min }}})}{({X}_{{\rm{\max }}}-{X}_{{\rm{\min }}})}$$

where ${X^{\prime} }_{i}$ is the normalized data for parameter *i*; *X*_*i*_ is the original data for parameter *i*; *X*_*max*_ is the maximum of the corresponding parameter *i*; and *X*_*min*_ is the minimum of the corresponding parameter *i*.

### Wavelet transform coherence

Wavelet transform coherence (WTC) is a method for analyzing the coherence and phase lag between two time series as a function of both time and frequency. This method was implemented through the WaveletComp package provided by R (https://www.r-project.org/). It can be defined as follows:

8 $${R}_{xy}^{2}=\frac{{|S({W}_{xy})|}^{2}}{S({|{W}_{x}|}^{2})S({|{W}_{y}|}^{2})}$$

where *S* is the smoothing operator; *W*_*x*_ is continuous wavelet transform of time series *x*(*t*); *W*_*y*_ is continuous wavelet transform of time series *y*(*t*); *W*_*xy*_ is the cross-wavelet power of two time series *x*(*t*) and *y*(*t*).

The cross-wavelet power *w*_*xy*_ between two time series (*x*(*t*), *y*(*t*)) is defined as follows:

9 $${W}_{xy}={W}_{x}{W}_{y}^{\ast }$$

where *W*_*x*_ is continuous wavelet transform of time series *x*(*t*); ${W}_{y}^{\ast }$ is the complex conjugate of continuous wavelet transform of time series *y*(*t*).

### Analysis of relationship between NDVI and meteorological parameters

The Pearson correlation coefficients between the NDVI and the meteorological parameters were calculated to analyze the relationships between NDVI and meteorological parameters. Considering the effects of interactions between air temperature, precipitation and net radiation on the NDVI, the partial correlation coefficients between the NDVI and meteorological variables were also calculated to determine the main meteorological driving factors that affect NDVI variations. The formula of the Pearson correlation coefficient can be written as follows:

10 $${P}_{X,Y}=\frac{{\rm{cov}}(X,Y)}{{\sigma }_{X}{\sigma }_{Y}}=\frac{E[(X-{\mu }_{X})(Y-{\mu }_{Y})]}{{\sigma }_{X}{\sigma }_{Y}}$$

where *P*_*X,Y*_ is the Pearson correlation coefficient between *X* and *Y*; cov (*X*, *Y*) is the covariance between *X* and *Y*; *σ*_*x*_ is the standard deviation for *X*; *σ*_*Y*_ is the standard deviation for *Y*; *μ*_*x*_ is the mean of *X*; and *μ*_*Y*_ is the mean of *Y*.

### Calculation of time lags between NDVI and meteorological parameters

The meteorological and NDVI data were collected in 10-day intervals (i.e., January 1 to 10 is the 1^st^ time unit; December 20 to 31 is the 36^th^ time unit). Taking spring as an example to analyze time lags of the NDVI response to climate parameters, a correlative coefficient was calculated using the meteorological data (from the 7^th^ time unit to the 15^th^ time unit) combined the NDVI data (from the 7^th^ time unit to the 15^th^ time unit). Thereafter, the previous meteorological factor duration set (from the 6^th^ time unit to the 14^th^ time unit) was combined with the NDVI dataset (still from the 7^th^ time unit to the 15^th^ time unit) to calculate the second correlative coefficient, and so on. Finally, time lags were determined using Eq. (11):

11 $$\rho =\,\max \,[{\rho }_{0},{\rho }_{1},{\rho }_{2},{\rho }_{3},\,\mathrm{.....},\,{\rho }_{i}]$$

where *ρ* is the lag correlation coefficient; and *ρ*_*i*_ is the Pearson correlation coefficient between NDVI and meteorological parameters of *i* times ten days earlier than the NDVI time unit. If *ρ* = *ρ*_*i*_ (*i* = 0, 1, 2, 3, 4, ….., 9), then time lags are 0, 10, 20, 30, 40, ……, 90 days, accordingly.

## Data Availability

The datasets generated and analyzed during the current study are available from the corresponding author on reasonable request.
